# A Flow Cytometry-Based Whole Blood Natural Killer Cell Cytotoxicity Assay Using Overnight Cytokine Activation

**DOI:** 10.3389/fimmu.2020.01851

**Published:** 2020-08-14

**Authors:** Jinho Kim, Minh-Trang Thi Phan, SoonHo Kweon, HongBi Yu, Jeehun Park, Kyeong-Hee Kim, Ilwoong Hwang, Sangbin Han, Min-Jung Kwon, Duck Cho

**Affiliations:** ^1^Department of Health Sciences and Technology, SAIHST, Sungkyunkwan University, Seoul, South Korea; ^2^Samsung Medical Center, Stem Cell & Regenerative Medicine Institute, Seoul, South Korea; ^3^Research Institute of Advanced Materials, Seoul National University, Seoul, South Korea; ^4^Department of Laboratory Medicine, Dong-A University College of Medicine, Busan, South Korea; ^5^Department of Emergency Medicine, Konkuk University Chungju Hospital, Chungju, South Korea; ^6^Department of Anesthesiology and Pain Medicine Samsung Medical Center, Sungkyunkwan University School of Medicine, Seoul, South Korea; ^7^Department of Laboratory Medicine, Kangbuk Samsung Hospital, Sungkyunkwan University School of Medicine, Seoul, South Korea; ^8^Department of Laboratory Medicine and Genetics, Samsung Medical Center, Sungkyunkwan University School of Medicine, Seoul, South Korea

**Keywords:** natural killer cells, activity assay, cytotoxicity, whole blood, cytokines

## Abstract

**Background:** Measurement of natural killer (NK) cell function has important clinical utility in several diseases. Although the flow cytometry (FC)-based 4-h NK cytotoxicity assay using peripheral blood mononuclear cells (PBMCs) in the clinical laboratory has been used for this purpose, this assay requires large amounts of blood and a rapid PBMC isolation step. Here, we developed an FC-based overnight NK cytotoxicity assay using whole blood (WB), and applied it to patients with liver diseases.

**Methods:** Peripheral blood of healthy volunteers (*n* = 28) and patients with liver diseases, including hepatocellular carcinoma (*n* = 19) and liver cirrhosis (*n* = 7), was analyzed for complete blood count, absolute NK cell count, and NK cell activity (NKA). NKA was evaluated in three assay types: an FC-based overnight WB NK cytotoxicity assay using carboxyfluorescein diacetate succinimidyl ester-labeled K562 cells in the presence of various cytokine combinations [including interleukin (IL)-2, IL-18, and IL-21], an FC-based 4-h PBMC NK cytotoxicity assay, and an FC-based CD107a degranulation assay using WB and PBMCs.

**Results:** Optimal cytokine combinations for NK cell activation in WB were determined (IL-2/IL-18, IL-2/IL-21, and IL-2/IL-18/IL-21). A good correlation was observed between WB and PBMC NK cytotoxicity assays; absolute NK cell counts were better correlated with the WB NK cytotoxicity assay than with the PBMC NK cytotoxicity assay. This WB NK cytotoxicity assay showed that patients with liver diseases had significantly lower NK cytotoxicity than healthy volunteers, under stimulation with various cytokines (*p* < 0.001).

**Conclusion:** The proposed FC-based overnight WB NK cytotoxicity assay correlates well with the conventional 4-h PBMC NK cytotoxicity assay, demonstrating future potential as a supportive assay for clinical laboratory research and observational studies.

## Background

Natural killer (NK) cells, one of the innate immune cell types, perform important roles in pathogens defense and participate in immune surveillance and cancer cell elimination (1). The cell surface density of CD56 and CD16 can be used to classify functionally distinct NK cell subsets: the CD56^bright^ CD16^−^subset (~10%), which secretes cytokines such as interferon-γ, activates the immune response, and the CD56^dim^ CD16^+^ subset (~80–90%), which directly lyses its targets (2, 3). Impaired NK cell activity (NKA) is highly correlated with several diseases. Particularly, low or absent NKA is a major characteristic of hemophagocytic lymphohistiocytosis (HLH). Thus, NKA has been used as a marker for HLH diagnosis (4–6). Low NKA has also been reported in various types of cancers (7, 8). Hepatocellular carcinoma (HCC) is an example of an NK cell-related disease, and the need to use low NKA as a marker for diagnosing HCC has been argued in several studies (9, 10). Therefore, there exists an increased demand for effective and relevant NKA measurement methods that are sensitive, precise, and convenient.

NKA can be classified into two major types: cytotoxicity and cytokine release, both of which are important for immune response (11, 12). Cytotoxicity, which refers to the killing of abnormal cells or tumor cells via the release of cytotoxic granules, including perforin and granzyme, can be primarily measured using a CD107a degranulation assay or flow cytometry (FC)-based NK cytotoxicity assay (13, 14). The CD107a assay measures the amount of secreted cytotoxic granules, which trigger target cell death. Therefore, the CD107a assay is used to measure NK cytotoxicity, but is an indirect measurement method. The FC-based NK cytotoxicity assay measures the killed target ratio, which can be considered a more convincing indicator of the killing ability of NK cells. Cytokine release [for example, the secretion of interferon gamma (IFN-γ)], the other type of NKA, differs from cytotoxicity. Cytokine release activity can be measured by FC via intracellular staining of the cytokines of interest or the enzyme-linked immunosorbent assay (ELISA) (15). Hence, depending on the purpose, NKA should be measured using an appropriate method for diagnosis and to understand the role of NK cells in diverse diseases.

There are some considerations for measuring NKA for diagnostic use in the clinical laboratory: ideally, assays should be non-biohazardous and easy to perform, requiring a small volume of blood. The ^51^Cr release assay is not preferred as it involves the use of a biohazardous radioactive isotope; instead, an FC-based peripheral blood mononuclear cell (PBMC) NK cytotoxicity assay is commonly used (4, 5, 11). However, this assay requires a large volume of blood and involves a PBMC isolation step. Therefore, whole blood (WB) is preferred for NKA measurement. In addition, the assay of NKA using WB reflects the *in vivo* state in a better manner (16, 17).

However, the WB cytotoxicity assay evaluates NK cytotoxicity using the Cr^51^ release assay, which uses a radio-reactive material. Recently, the ELISA-based measurement of the IFN-γ amount secreted from NK cells in WB stimulated with a specific cytokine combination has also been used in diagnosis and NK cell study (18–21). However, this assay is not ideal for measuring NK cytotoxicity and has not been studied in correlation with the NK cytotoxicity assay. Thus, the development of a convenient method of the WB NK cytotoxicity assay is necessary for clinical laboratory research.

In this study, we developed an FC-based overnight WB NK cytotoxicity assay via cytokine activation and compared it to an established FC-based 4-h PBMC NK cytotoxicity assay. To investigate the potential value for the clinical use of this assay, we compared the NKA of patients with liver diseases (HCC and liver cirrhosis) with that of healthy individuals.

## Materials and Methods

### Blood Sample Preparation

Peripheral blood was collected in heparinized tubes from 28 healthy volunteers (15 men and 13 women, with ages ranging from 28 to 53). All donors provided written informed consent prior to study participation. To investigate clinical applicability, we collected blood samples from patients with liver diseases [*n* = 26; HCC (*n* = 19) and liver cirrhosis (*n* =7)] to evaluate NK cell activity utilizing the overnight WB NK cytotoxicity assay. This study was approved by the Institutional Review Board of the Samsung Medical Center, Seoul, Korea (IRB No. SMC 2018-11-005-004). Peripheral blood was used for determining the complete blood count (CBC), and for performing the CD107a degranulation assay and the NK cytotoxicity assay using WB and PBMCs. The CBC was measured on a Sysmex XE-2100 analyzer (Sysmex, Kobe, Japan). Human PBMCs were isolated from healthy adult donors using density-gradient centrifugation with Ficoll-Hypaque (d = 1.077, LymphoprepTM; Axis-Shield, Oslo, Norway) and washed twice with phosphate-buffered saline (PBS) (Welgene, Gyeongsan-si, Gyeongsangbuk-do, Korea).

### Cells and Cell Culture

Human myelogenous leukemia (K562) cells were obtained from the American Type Culture Collection (ATCC, Manassas, VA, USA) and cultured in Roswell Park Memorial Institute (RPMI) 1640 medium supplemented with 10% heat-inactivated fetal bovine serum (Gibco, Grand Island, NY, USA), 100 U/mL penicillin, and 100 μg/mL streptomycin (Lonza, Walkersville, MD, USA) at 37°C in a humidified 5% CO_2_ incubator.

### Cytokines and Antibodies

Recombinant human interleukin (IL)-2, IL-21 (PeproTech, Rocky Hill, NJ, USA), and IL-18 (MBL International, Woburn, MA, USA) were used to stimulate NK cells in WB samples. Fluorescein isothiocyanate (FITC)-conjugated anti-human CD3 monoclonal antibody (mAb) and phycoerythrin (PE)-cyanine (Cy)5-conjugated anti-human CD56 mAb were used to evaluate NK cell purity; PE-conjugated anti-human CD107a mAbs were used as markers of degranulation. All fluorescent mAbs were obtained from BD Biosciences (San Jose, CA, USA).

### NK Cell Proportion Analysis

Peripheral WB samples (100 μL) were incubated with CD3 and CD56 mAb for 15 min at room temperature in the dark. Red blood cells (RBCs) were lysed by the addition of 2 mL fluorescence-activated cell sorting (FACS) Lysing Solution (BD Biosciences) for 10 min and washed with PBS. Cells were run on a FACS Canto II FC (BD Biosciences); 30,000–50,000 events were acquired in lymphocyte gate. Analysis was performed using Kaluza software version 1.3 (Beckman Coulter, Brea, CA, USA). The absolute NK cell count was calculated by multiplying the total number of lymphocytes by the percentage of CD3^−^CD56^+^ cells determined by FC.

### CD107a Degranulation Assay

To analyze NK cell degranulation, their CD107a expression was measured as previously described (13, 17). WB (1 mL) or 1 × 10^6^ PBMCs were mixed with 1 mL of RPMI medium containing various cytokine combinations [untreated; IL-2 (200 U/mL); IL-2 (200 U/mL) + IL-18 (50 ng/mL); IL-2 (200 U/mL) + IL-21 (10 ng/mL); and IL-2 (200 U/mL) + IL-18 (50 ng/mL) + IL-21 (10 ng/mL)]. Mixed WB (200 μL) or 2 × 10^5^ PBMCs was incubated in FACS tubes with 5 μL PE-conjugated anti-CD107a mAb in the absence or presence of 2 × 10^5^ K562 cells. Monensin (2 μM; eBiosciences) was added following a 1 h incubation, and cells were incubated for an additional 2 h. Thereafter, cells were stained with FITC-conjugated anti-human CD3 mAb and PE-Cy5-conjugated anti-human CD56 mAb for 15 min at 4°C in the dark, followed by RBC lysis using 2 mL of FACS Lysing Solution. Cells were subsequently washed with PBS and analyzed on a FACSVerse FC (BD Biosciences). The gating strategy for analyzing CD107a expression on NK cells is described in **Figure 2A**. PBMCs or WB were also incubated with cytokines overnight (16–22 h) at 37°C in a humidified 5% CO_2_ incubator, and then CD107a assays were performed as described above.

### PBMC NK Cytotoxicity Assay

The PBMC NK cytotoxicity assay using K562 cells was performed as previously described (22, 23). K562 target cells were labeled with carboxyfluorescein diacetate succinimidyl ester (CFSE; Life Technologies) for 10 min at 37°C in the dark and washed twice with complete media to quench the labeling reaction. CFSE-stained K562 cells (1 × 10^4^) were co-incubated with various effector cells (PBMCs) at 10:1, 20:1, and 40:1 effector:target (E:T) ratios for 4 h at 37°C in a humidified 5% CO_2_ incubator. Thereafter, mixed cells were transferred to FACS tubes and 1 μL of 1 mg/mL propidium iodide (PI) was added; subsequently, cells were run on a FACSVerse FC and analyzed using Kaluza software.

### WB NK Cytotoxicity Assay

**Figure 1:**
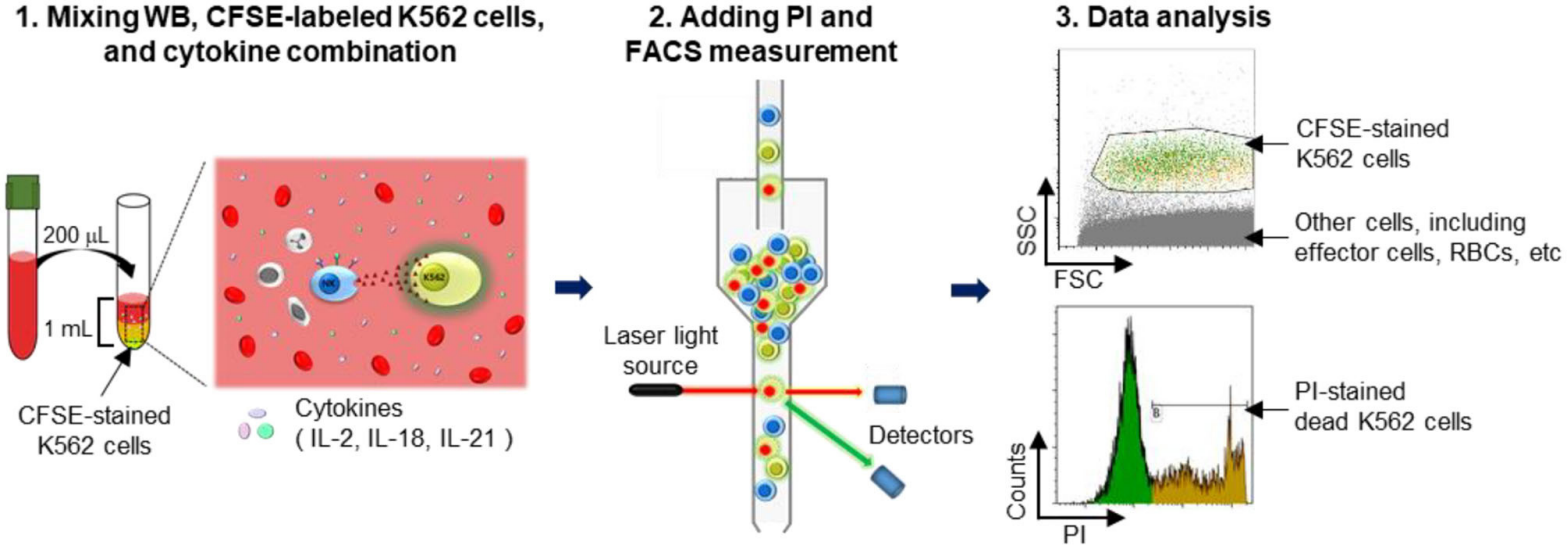
Schematic illustration of the experimental procedure. A detailed explanation is provided. Briefly, whole blood (WB) was mixed overnight with carboxyfluorescein succinimidyl ester (CFSE)-labeled K562 cells in media containing cytokines. Thereafter, propidium iodide (PI) was added to detect dead cells, and the killed target cell ratio was determined using flow cytometry.

K562 cells were stained with 1 μM/mL CFSE for 10 min at 37°C in a humidified 5% CO_2_ incubator and subsequently washed twice with RPMI 1640 medium. WB samples (50, 100, and 200 μL) were incubated with various cytokine combinations (100 U/mL IL-2; 100 U/mL IL-2 + 25 ng/mL IL-18; 100 U/mL IL-2 + 5 ng/mL IL-21; and 100 U/mL IL-2 + 25 ng/mL IL-18 + 5 ng/mL IL-21) and with 5 × 10^4^ CFSE-stained K562 cells in FACS tubes overnight (16–22 h) at 37°C in a humidified 5% CO_2_ incubator. Just prior to acquisition (5–10 min), 5 μL of 1 mg/mL PI (Sigma Aldrich, St. Louis, MO, USA) was added to each tube. Cells were run on a FACS Canto II FC and analyzed using Kaluza software. Percentages of dead target cells positive for CFSE and PI were calculated after subtracting the percentage of spontaneous death in target cells. A threshold was set on the FITC channel to ensure analysis of CFSE-labeled target cells; all other cells, including RBCs, were excluded (Figure 1). Assay controls used to define cell populations included K562 cells only (spontaneous lysis) and K562 cells with 100 μL of 100% ethanol (maximum lysis). Percent lysis of K562 target cells was calculated as [(percent experimental lysis—percent spontaneous lysis)/(percent maximum lysis—percent spontaneous lysis)] × 100.

### Statistical Analysis

Statistical analysis was performed with GraphPad Prism 5 software (GraphPad Software, San Diego, CA, USA). Between-group differences were assessed using the Kruskal-Wallis test, followed by the Dunn's post analysis test to define the significance between pairs of groups. The Mann-Whitney *U*-test (non-parametric) was used to analyze specific sample pairs for significant differences. All tests were two-tailed, and differences were considered statistically significant when *p* < 0.05.

## Results

### Selection of the Optimal Combination of NK Cell-Stimulating Cytokines by the CD107a Assay

**Figure 2:**
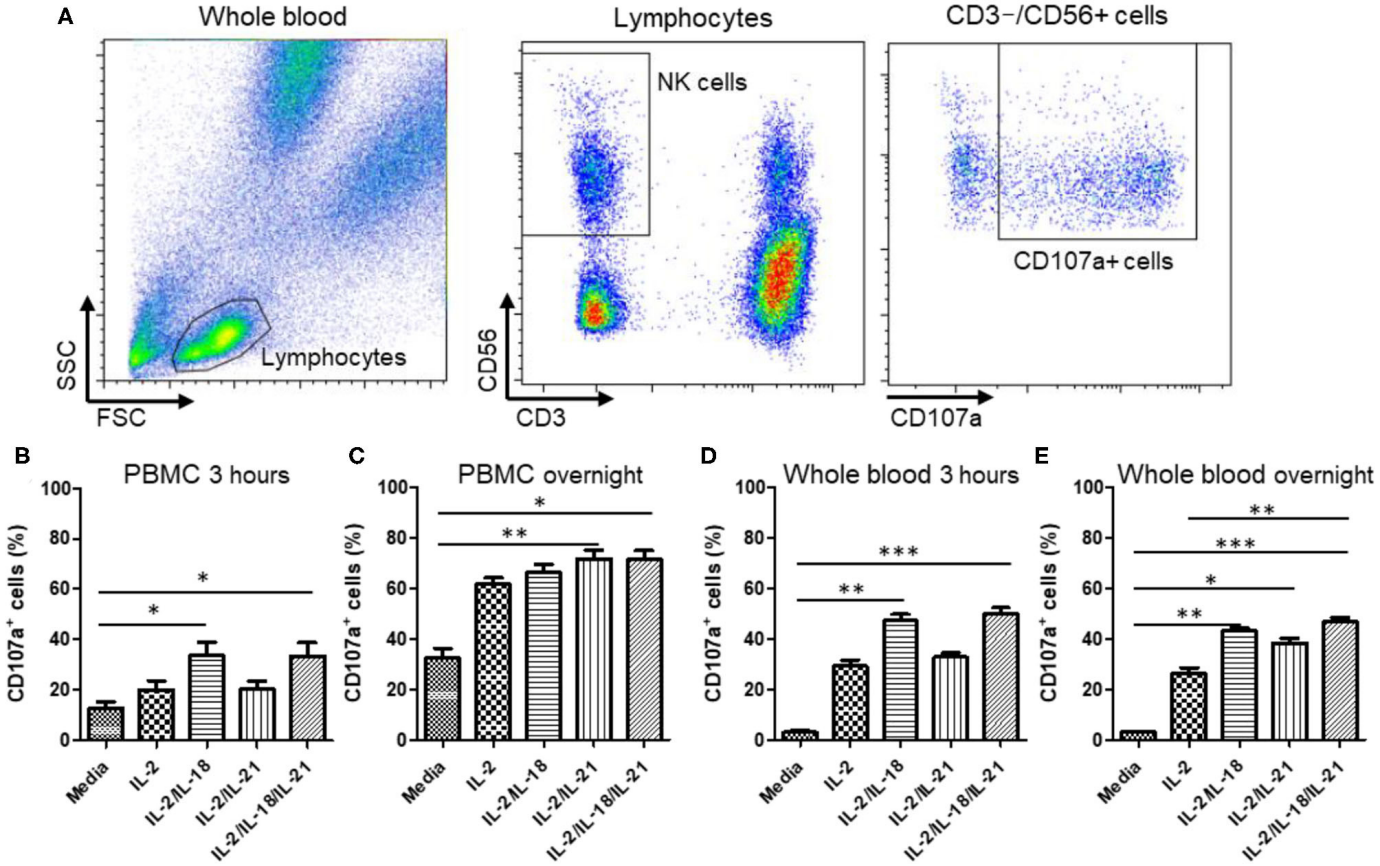
CD107a expression of peripheral blood mononuclear cells (PBMCs) and whole blood (WB) under various cytokine combinations and incubation times. PBMCs or WB samples were mixed with media containing various cytokine combinations (no cytokines (media), IL-2, IL-2/IL-18, IL-2/IL-21, and IL-2/IL-18/IL-21) for the indicated durations of time, and CD107a assays were performed either immediately or following overnight incubation. Samples were incubated for 3 h with or without K562 cells. **(A)** Gating strategy of CD107a measurement. CD107a surface expression was determined as CD3^−^CD56^+^CD107a^+^ by flow cytometry. **(B,C)** Bar graphs indicate the percentage of CD107a^+^ NK cells in PBMCs following cytokine stimulation for 3 h or overnight. **(D,E)** Bar graphs indicate percentages of CD107a^+^ NK cells in WB following cytokine stimulation for 3 h and overnight. Data are represented as the mean ± SEM of results from five donors (*p < 0.05; **p < 0.01; ***p < 0.001).

To select an optimal cytokine combination for the activation of NK cells in PBMCs and WB, we used a CD107a degranulation assay in five healthy donors. A significant increase in CD107a expression was observed when NK cells in both PBMC and WB samples were stimulated with either IL-2/IL-18 or IL-2/IL-18/IL-21 for 3 h [*p* = 0.016 and *p* = 0.008, respectively (PBMCs) and *p* = 0.008 and *p* = 0.0003, respectively (WB)] but not after treatment with IL-2 or IL-2/IL-21 (Figures 2B,D). Compared with the 3-h stimulation, overnight stimulation of NK cells in PBMC and WB samples led to markedly increased CD107a degranulation with all cytokine combinations (Figures 2C,E). These results suggest that NK cells in WB treated with the different cytokine combinations are effectively stimulated for use in the NK cytotoxicity assay.

### Overnight WB and 4-h PBMC NK Cytotoxicity Assays Provide Similar Results and Show Good Correlation

**Figure 3:**
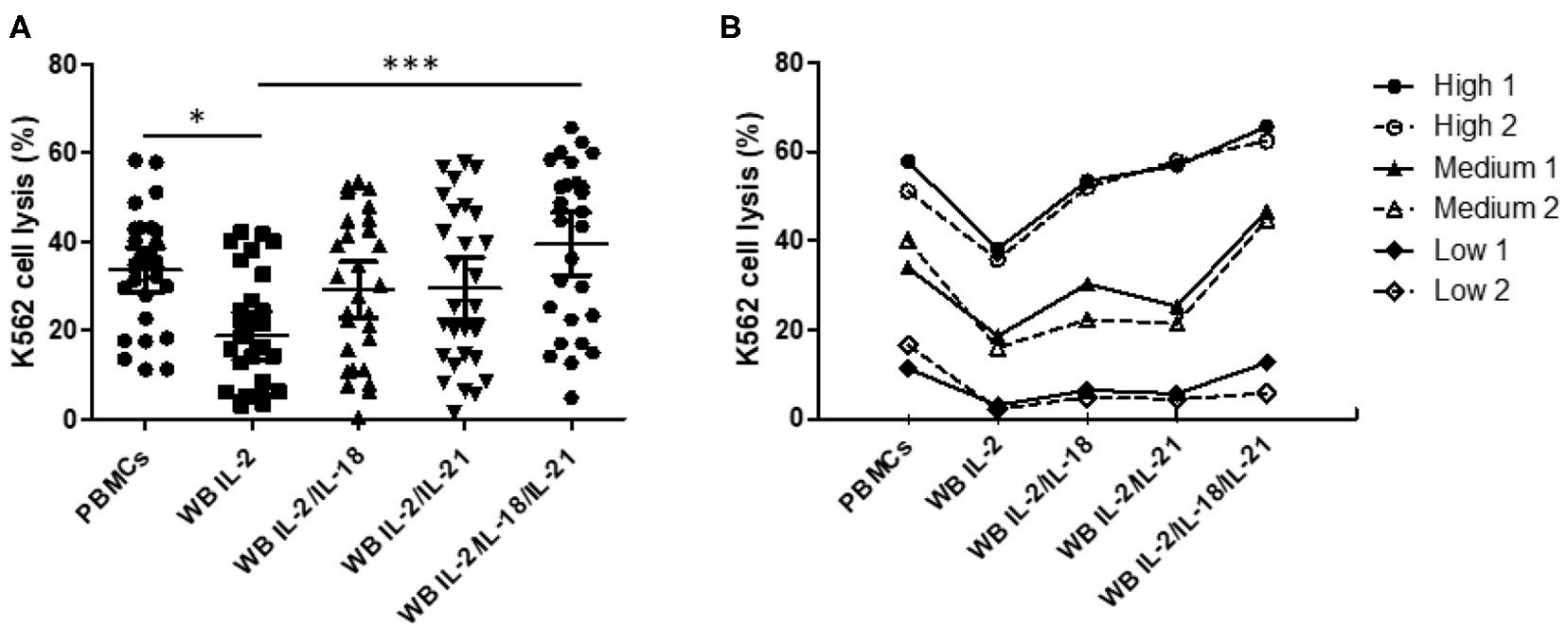
Comparison of NK cytotoxicity between the 4-h peripheral blood mononuclear cell (PBMC) assay without cytokine stimulation and the overnight whole blood (WB) assay with multiple cytokine combinations. PBMCs or WB were co-incubated with K562 cells, with various cytokine combinations added to WB, for 4 h (PBMCs) or overnight (WB). PBMC assays were performed at an E:T ratio of 40:1; WB assays used 200 μL WB. **(A)** Scatter plots of NK cytotoxicity in PBMCs and WB samples from 28 donors. Data are represented as the mean ± SEM (*p < 0.05; ***p < 0.001). **(B)** Representative data from two donors each with low, medium, and high NK cell cytotoxicity.

We confirmed increased CD107a expression upon treatment with different cytokine combinations. Based on the selected optimal cytokine combinations, we developed an FC-based overnight WB NK cytotoxicity assay using cytokine activation in 28 healthy donors. Overnight WB NK cells activated with IL2 displayed lower cytotoxicity than those in the 4-h PBMC NK cytotoxicity assay, whereas overnight WB NK cells activated by all other combinations (IL-2/IL-18, IL-2/IL-21, and IL-2/IL-18/IL-21) demonstrated similar cytotoxicity. Among these, the IL-2/IL-18/IL-21-activated WB NK cells had the highest cytotoxicity (Figure 3A). We confirmed that three kinds of donors with low, medium, and high NK cytotoxicity in the PBMC assay demonstrated the same pattern of cytotoxicity in the WB assay, and this pattern was not disturbed by the type of cytokine used for treatment (Figure 3B). In our preliminary study, we also compared the overnight PBMC NK cytotoxicity assay (E:T ratio of 4:1) with the overnight WB NK cytotoxicity assay (200 μL) and observed a good correlation between the assays using the same cytokine stimulation (i.e., IL-2/IL-18) (data not shown).

**Figure 4:**
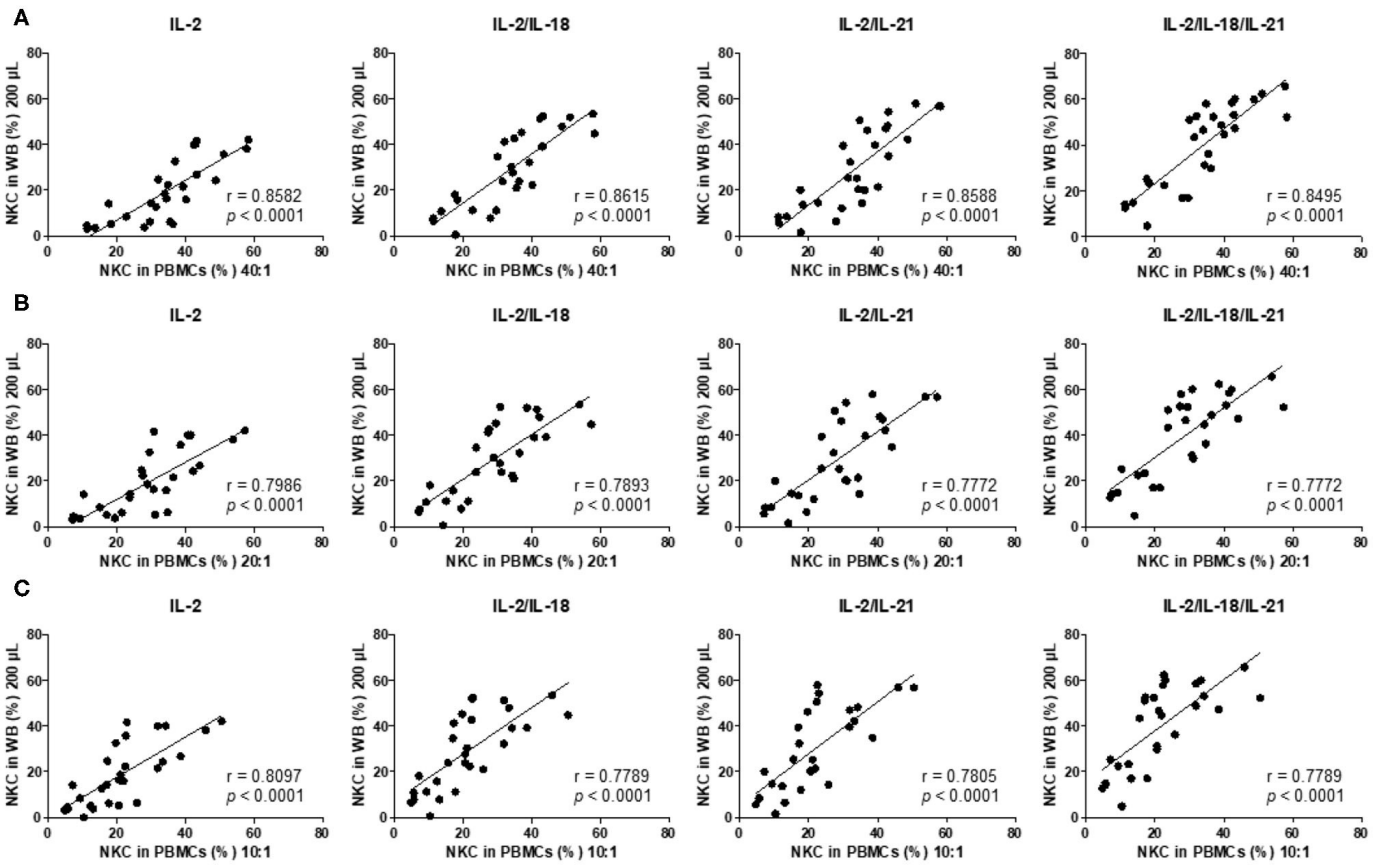
NK cytotoxicity correlations between peripheral blood mononuclear cells (PBMCs) and overnight cytokine-activated whole blood (WB) assays. Natural killer (NK) cytotoxicity assays were performed using PBMCs at an E:T ratio of **(A)** 40:1, **(B)** 20:1, and **(C)** 10:1 and 200 μL WB (*n* = 28 donors). Correlation between PBMCs and IL-2-, IL-2/IL-18-, IL-2/IL-21-, and IL-2/IL-18/IL-21-treated WB.

Thereafter, we analyzed the correlation between the results of the 4-h PBMC NK cytotoxicity assay (E:T ratio of 40:1, 20:1, and 10:1), which has been commonly used in clinical laboratories (4, 5), and the overnight WB NK cytotoxicity assay (200 μL) and observed significant positive linear relationships between the assays using multiple cytokine combinations (IL-2, IL-2/IL-18, IL-2/IL-21, and IL-2/IL-18/IL-21) (Figures 4A–C). Thus, we conclude that the overnight WB NK cytotoxicity assay using multiple cytokine stimulations can replace the commonly used 4-h PBMC NK cytotoxicity assay.

### Absolute NK Cell Count Correlates With Results of the Overnight WB NK Cytotoxicity Assay

**Figure 5:**
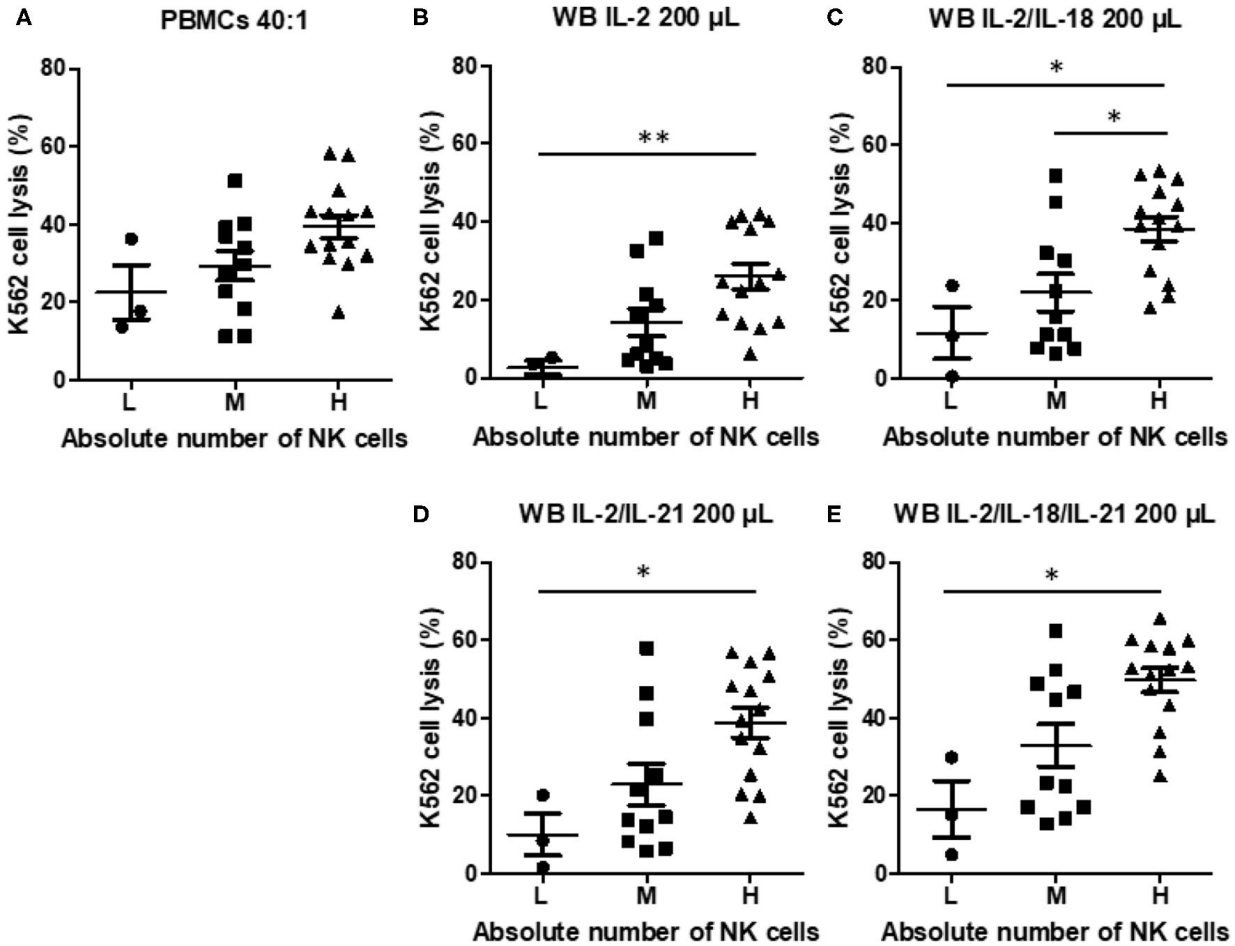
Natural killer (NK) cytotoxicity measured by peripheral blood mononuclear cells (PBMC) NK cytotoxicity (40:1) and whole blood (WB)-based NK cytotoxicity (200 μL) according to the absolute number of NK cells (low, three donors; medium, 11 donors; high, 14 donors). NK cytotoxicity results using **(A)** PBMCs and WB activated overnight with **(B)** IL-2, **(C)** IL-2/IL-18, **(D)** IL-2/IL-21, and **(E)** IL-2/IL-18/IL-21. Data are represented as the mean ± SEM (*p < 0.05; **p < 0.01).

To examine the effect of the absolute NK cell count on NK cytotoxicity when using PBMCs or WB, we classified the samples into three groups: low (L, *n* = 3), medium (M, *n* = 11), and high (H, *n* = 14), on the basis of the absolute NK cell count. NK cytotoxicity in PBMCs appeared to increase with an increase in the absolute NK cell count, but the increase was not significantly different (Figure 5A). However, NK cytotoxicity in WB stimulated with cytokines displayed a significant difference depending on the absolute NK cell count (Figures 5B–E). These data suggest that the absolute NK cell count correlates more with the results of the overnight WB NK cytotoxicity assay than with those of the PBMC NK cytotoxicity assay.

### NKA Was Impaired in Patients With Liver Cancer

**Figure 6:**
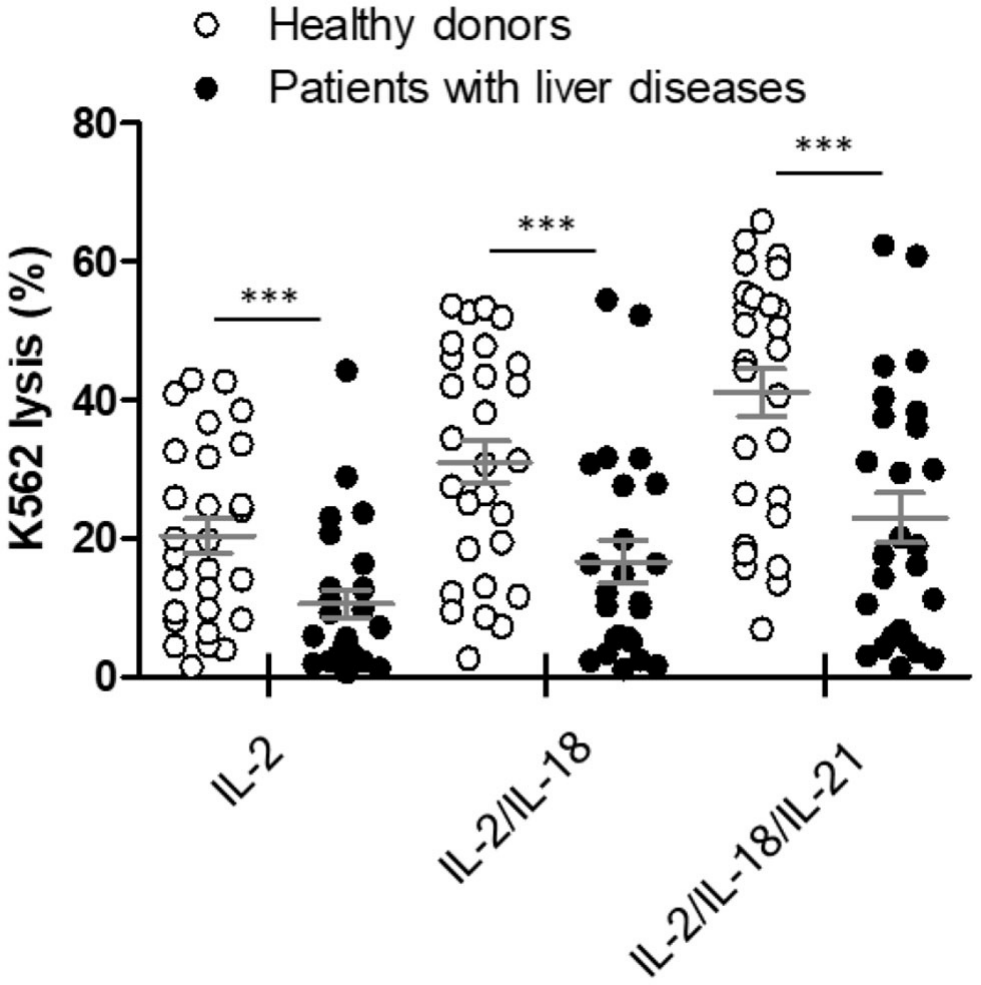
Comparison of natural killer (NK) cytotoxicity in healthy donors (*n* = 28) and patients with liver diseases (*n* = 26) using the whole blood (WB)-based NK cytotoxicity assay (200 μL). WB was activated overnight with IL-2, IL-2/IL-18, and IL-2/IL-18/IL-21. Data are represented as the mean ± SEM (***p < 0.001).

The NK cytotoxicity between heathy donors (*n* = 28) and patients with liver diseases (*n* = 26) was compared using our WB-based NK cytotoxicity assay (200 μL WB: 5 × 10^4^ K562 cells). Patients with liver diseases demonstrated considerably lower NK cytotoxicity than healthy donors under all cytokine stimulations (Figure 6). No significant differences in NK cytotoxicity were observed between HCC and liver cirrhosis patients (Figure S1). Therefore, our data suggest that this overnight WB NK cytotoxicity assay using cytokine treatment could be utilized as a supportive tool for diagnosis, or to understand the role of NK cells in patients with liver diseases.

## Discussion

We developed an FC-based overnight WB NK cytotoxicity assay involving cytokine (IL-2/IL-18, IL-2/IL-21, and IL-2/IL-18/IL-21) activation, and demonstrated that it has a good correlation with an established 4-h PBMC NK cytotoxicity assay. A positive correlation was observed between the absolute NK cell count and the NK cytotoxicity determined using the WB NK cytotoxicity assay, but not the PBMC NK cytotoxicity assay. Finally, we measured the NK cytotoxicity of healthy donors and patients using our method; patients with liver diseases demonstrated a considerably lower NK cytotoxicity than healthy donors under all cytokine stimulations.

Compared with PBMC-based assays, WB-based assays are simple convenient, requiring smaller amounts of blood. Therefore, several WB-based NKA assays measuring CD107a degranulation and cytokine production (analysis of intracellular IFNγ using FC or secreted IFNγ using ELISA) have been developed (15, 24, 25). However, these assays do not directly measure the cytotoxicity of NK cells. In this study, we developed an FC-based WB NK cytotoxicity assay to evaluate the ability of NK cells to directly kill target cells.

Recently, Mhatre et al. proposed a rapid, FC-based, WB cytotoxicity assay for the evaluation of NK cell function. They compared NK cytotoxicity against 3,3′-dioctadecyloxacarbocyanine perchlorate-labeled K562 cells in PBMC and WB samples from healthy individuals and patients with familial HLH without cytokine stimulation (26). However, we could not reproduce their assay, and no other group has demonstrated the reproducibility of their assay (data not shown). We speculate that this is attributed to the relatively low number of NK cells with low activation levels in WB.

There are several cytokine candidates for activating NK cells in WB. IL-2 and IL-15 have been reported to stimulate NK cell proliferation and enhance NK cytotoxicity (27). Claus et al. described a rapid, comprehensive, WB-based FC assay using IL-2 or IL-12/IL-18 stimulation (17). A synergistic effect on NK cell activation has been observed upon stimulation with IL-2 together with IL-18 or IL-21 (24, 28, 29). Moreover, IL-21 synergizes with IL-15 or IL-18 to enhance IFNγ production in human NK and T cells (30). NKvue, one of the most widely used WB-based NKA assays with an ELISA-based IFN-γ measurement, also requires a recombinant cytokine combination called Promoca for stimulating NK cells; however, the detailed cytokine components of Promoca are not disclosed (15). In this study, we identified optimal cytokines using the CD107a assay, which is commonly used in diverse NK cell studies. Notably, even under conditions of no cytokine treatment, the overall CD107a expression in PBMCs, but not WB, was increased. This finding might be attributed to the effect of granulocytes, such as neutrophils, that can reduce NK cytotoxicity. Thus, PBMCs, with no granulocytes, can be activated to a greater extent by endogenous cytokine production (31).

In the clinical laboratory, FC-based PBMC NK cytotoxicity assays are commonly performed (4, 5, 11). The widely used PBMC-based assays require a relatively large volume of peripheral blood (obtaining such a volume is difficult in pediatric patients or patients with lymphocytopenia) and involve laborious isolation procedures, including centrifugation, washing, and counting of PBMCs. By contrast, our WB assay requires only 200 μL of WB and involves a simple measuring procedure: mixing WB with labeled target cells in media containing cytokine combinations. Moreover, our overnight WB NK cytotoxicity assay via cytokine stimulation has good correlation and comparable sensitivity with the 4-h PBMC NK cytotoxicity assay. In an attempt to explain the comparable NK cell cytotoxicity of cytokine-stimulated WB samples and that of unstimulated PBMCs, we analyzed the expression of various NK cell receptors (CD16, NKG2A, NKG2C, NKG2D, CD57, CD8a, NKp30, NKp46, CD62L, and DNAM-1) in NK cells of WB samples, before and after overnight cytokine stimulation. We found that NKp46 expression was significantly greater in cytokine-stimulated NK cells compared with unstimulated NK cells, whereas expression of the other receptors remained unchanged (data not shown). It is well-known that natural cytotoxic receptors (including NKp46) are associated with the cytotoxic capabilities of NK cells (32–34). The increase in NKp46 expression might be a response to the cytokines, but further studies are necessary to clarify this issue.

Regarding the correlation between the NK cell count and NKA, some studies have reported a positive correlation, whereas others have reported a poor correlation (35–37). We found extremely low NK cytotoxicity in patients with low white blood cell counts using the WB assay, but relatively high NK cytotoxicity using the PBMC assay. The WB assay appeared to be more dependent on the number of NK cells in comparison to the PBMC assay. It is possible that PBMCs contain a relatively higher number of NK cells than WB owing to the isolation process and adjustment of the E:T ratio, which can introduce errors, especially in patients with low or high NK cell counts. As a result, our WB assay might better reflect the actual NK cell function in the body than the PBMC assay.

The lower NK cytotoxicity in cancer-related diseases is well-known. Our study demonstrated low NK cytotoxicity (measured using this WB assay) in patients with HCC and liver cirrhosis, consistent with the results of previous studies (38–40). Our data suggest that this WB-based NK cytotoxicity assay has potential for further development as a supportive diagnostic tool for these severe liver diseases.

We were unable to compare the NKA measured by our new WB-based assay with previously used WB-based assays such as NKvue. Although both assays measure different NK cell activities, cytotoxicity, and cytokine release, respectively, the comparison might be useful to guide proper NKA measurement.

In conclusion, we described a simple and easily reproducible method to evaluate NK cytotoxicity in a small amount of WB, where the amount of available blood may be limited. This method has the potential for application in clinical laboratory research and observational studies.

## Data Availability Statement

All datasets generated for this study are included in the article/Supplementary Material.

## Ethics Statement

This study was approved by the Institutional Review Board of the Samsung Medical Center, Seoul, Korea (IRB No. SMC 2018-11-005-004). Peripheral blood was used for this study. The patients/participants provided their written informed consent to participate in this study.

## Author Contributions

JK and M-TP performed the research and analyzed data. SK, HY, JP, K-HK, and IH contributed to data analysis. M-JK and DC designed the research. JK, M-TP, JP, K-HK, M-JK, and DC wrote the paper. All authors contributed to the article and approved the submitted version.

## Conflict of Interest

The authors declare that the research was conducted in the absence of any commercial or financial relationships that could be construed as a potential conflict of interest.

## Supplementary Material

Figure S1 Comparison of natural killer (NK) cytotoxicity in hepatocellular carcinoma (*n* = 19, red) and liver cirrhosis (*n* = 7, blue) patients using whole blood (WB)-based NK cytotoxicity (200 μL). WB was activated overnight with IL-2, IL-2/IL-18, and IL-2/IL-18/IL-21. Data are represented as the mean ± SEM.

Table S1 Performance characteristics of the NK cytotoxicity assay based on PBMCs or WB.

## References

[1] Vivier E Raulet DH Moretta A Caligiuri MA Zitvogel L Lanier LL Innate or adaptive immunity? the example of natural killer cells. Science. (2011) 331:44–9. 10.1126/science.1198687 21212348 PMC3089969

[2] Caligiuri MA. Human natural killer cells. Blood. (2008) 112:461–9. 10.1182/blood-2007-09-077438 18650461 PMC2481557

[3] Chiorean EG Miller SJ. The biology of natural killer cells and implications for therapy of human disease. J Hematother Stem Cell Res. (2001) 10:451–63. 10.1089/15258160152509073 11522229

[4] Zhang J Wang Y Wu L Wang J Tang R Li S. Application of an improved flow cytometry-based NK cell activity assay in adult hemophagocytic lymphohistiocytosis. Int J Hematol. (2017) 105:828–34. 10.1007/s12185-017-2195-3 28185204

[5] Chung HJ Park CJ Lim JH Jang S Chi HS Im HJ. Establishment of a reference interval for natural killer cell activity through flow cytometry and its clinical application in the diagnosis of hemophagocytic lymphohistiocytosis. Int J Lab Hematol. (2010) 32:239–47. 10.1111/j.1751-553X.2009.01177.x 19614711

[6] Lee H Kim HS Lee JM Park KH Choi AR Yoon JH. Natural killer cell function tests by flowcytometry-based cytotoxicity and IFN-gamma production for the diagnosis of adult hemophagocytic lymphohistiocytosis. Int J Mol Sci. (2019) 20:5413. 10.3390/ijms20215413 31671661 PMC6862274

[7] Lee JC Lee KM Kim DW Heo SD. Elevated TGF-beta1 secretion and down-modulation of NKG2D underlies impaired NK cytotoxicity in cancer patients. J Immunol. (2004) 172:7335–40. 10.4049/jimmunol.172.12.7335 15187109

[8] Sutlu T Alici E. Natural killer cell-based immunotherapy in cancer: current insights and future prospects. J Intern Med. (2009) 266:154–81. 10.1111/j.1365-2796.2009.02121.x 19614820

[9] Sun C Sun HY Xiao WH Zhang C Tian GZ. Natural killer cell dysfunction in hepatocellular carcinoma and NK cell-based immunotherapy. Acta Pharmacol Sin. (2015) 36:1191–9. 10.1038/aps.2015.41 26073325 PMC4648180

[10] Liu P Chen L Zhang H. Natural killer cells in liver disease and hepatocellular carcinoma and the NK cell-based immunotherapy. J Immunol Res. (2018) 2018:1206737. 10.1155/2018/1206737 30255103 PMC6142725

[11] Wong WY Wong H Cheung SP Chan E. Measuring natural killer cell cytotoxicity by flow cytometry. Pathology. (2019) 51:286–91. 10.1016/j.pathol.2018.12.417 30803738

[12] Trotta R Ciarlariello D Dal Col J Neviani P Santhanam R Mao H. The PP2A inhibitor SET regulates natural killer cell IFN-gamma production. J Exp Med. (2007) 204:2397–405. 10.1084/jem.20070419 17875674 PMC2118465

[13] Aktas E Kucuksezer UC Bilgic S Erten G Deniz G. Relationship between CD107a expression and cytotoxic activity. Cell Immunol. (2009) 254:149–54. 10.1016/j.cellimm.2008.08.007 18835598

[14] Alter G Malenfant JM Altfeld M. CD107a as a functional marker for the identification of natural killer cell activity. J Immunol Methods. (2004) 294:15–22. 10.1016/j.jim.2004.08.008 15604012

[15] Lee SB Cha J Kim IK Yoon JC Lee HJ Park SW. A high-throughput assay of NK cell activity in whole blood and its clinical application. Biochem Biophys Res Commun. (2014) 445:584–90. 10.1016/j.bbrc.2014.02.040 24561245

[16] Dons'koi BV Chernyshov VP Osypchuk VD. Measurement of NK activity in whole blood by the CD69 up-regulation after co-incubation with K562, comparison with NK cytotoxicity assays and CD107a degranulation assay. J Immunol Methods. (2011) 372:187–95. 10.1016/j.jim.2011.07.016 21839083

[17] Claus M Greil J Watzl C. Comprehensive analysis of NK cell function in whole blood samples. J Immunol Methods. (2009) 341:154–64. 10.1016/j.jim.2008.11.006 19056395

[18] Kim BR Chun S Cho D Kim HK. Association of neutrophil-to-lymphocyte ratio and natural killer cell activity revealed by measurement of interferon-gamma levels in a healthy population. J Clin Lab Anal. (2019) 33:e22640. 10.1002/jcla.22640 30105845 PMC6430355

[19] Choi SI Lee SH Park JY Kim KA Lee EJ Lee SY. In: clinical utility of a novel natural killer cell activity assay for diagnosing non-small cell lung cancer: a prospective pilot study. Onco Targets Ther. (2019) 12:1661–9. 10.2147/OTT.S194473 30881021 PMC6398406

[20] Lee J Park KH Ryu JH Bae HJ Choi A Lee H. Natural killer cell activity for IFN-gamma production as a supportive diagnostic marker for gastric cancer. Oncotarget. (2017) 8:70431–40. 10.18632/oncotarget.19712 29050291 PMC5642566

[21] Kim JH Park K Lee SB Kang S Park JS Ahn CW. Relationship between natural killer cell activity and glucose control in patients with type. 2 diabetes and prediabetes. J Diabetes Invest. (2019) 10:1223–8. 10.1111/jdi.13002 30618112 PMC6717814

[22] Kandarian F Sunga GM Arango-Saenz D Rossetti M. A flow cytometry-based cytotoxicity assay for the assessment of human NK cell activity. J Vis Exp. (2017) 126:56191. 10.3791/56191 28829424 PMC5614136

[23] Kweon S Phan MT Chun S Yu H Kim J Kim S. Expansion of human NK cells using K562 cells expressing OX40 ligand and short exposure to IL-21. Front Immunol. (2019) 10:879. 10.3389/fimmu.2019.00879 31105701 PMC6491902

[24] Claus M Watzl C. Evaluation of human natural killer cell activities in whole blood. Curr Protoc Immunol. (2010) 7:Unit7.39. 10.1002/0471142735.im0739s91 21053306

[25] Jung YS Kwon MJ Park DI Sohn CI Park HJ. Association between natural killer cell activity and the risk of colorectal neoplasia. J Gastroenterol Hepatol. (2018) 33:831–6. 10.1111/jgh.14028 29055146

[26] Mhatre S Madkaikar M Ghosh K Desai M Pujari V Gupta M. Rapid flow cytometry based cytotoxicity assay for evaluation of NK cell function. Indian J Exp Biol. (2014) 52:983–8. 25345247

[27] Rocca YS Roberti MP Julia EP Pampena MB Bruno L Rivero S Phenotypic and functional dysregulated blood NK cells in colorectal cancer patients can be activated by cetuximab plus IL-2 or IL-15. Front Immunol. (2016) 7:413 10.3389/fimmu.2016.00413 27777574 PMC5056190

[28] Nielsen CM Wolf AS Goodier MR Riley ME. Synergy between common gamma chain family cytokines and IL-18 potentiates innate and adaptive pathways of NK cell activation. Front Immunol. (2016) 7:101. 10.3389/fimmu.2016.00101 27047490 PMC4801862

[29] Lim DP Jang YY Kim S Koh SS Lee JJ Kim JS. Effect of exposure to interleukin-21 at various time points on human natural killer cell culture. Cytotherapy. (2014) 16:1419–30. 10.1016/j.jcyt.2014.04.008 24950680

[30] Strengell M Matikainen S Siren J Lehtonen A Foster D Julkunen I. IL-21 in synergy with IL-15 or IL-18 enhances IFN-gamma production in human NK and T cells. J Immunol. (2003) 170:5464–9. 10.4049/jimmunol.170.11.5464 12759422

[31] Son BK Roberts RL Ank BJ Stiehm RE. Effects of anticoagulant, serum, and temperature on the natural killer activity of human peripheral blood mononuclear cells stored overnight. Clin Diagn Lab Immunol. (1996) 3:260–4. 10.1128/CDLI.3.3.260-264.1996 8705665 PMC170325

[32] Sivori S Pende D Bottino C Marcenaro E Pessino A Biassoni R NKp46 is the major triggering receptor involved in the natural cytotoxicity of fresh or cultured human NK cells. correlation between surface density of NKp46 and natural cytotoxicity against autologous, allogeneic or xenogeneic target cells. Eur J Immunol. (1999) 29:1656–66. 10.1002/(SICI)1521-4141(199905)29:05&lt;1656::AID-IMMU1656&gt;3.0.CO;2-1 10359120

[33] Phan MT Chun S Kim SH Ali AK Lee SH Kim S. Natural killer cell subsets and receptor expression in peripheral blood mononuclear cells of a healthy Korean population: reference range, influence of age and sex, and correlation between NK cell receptors and cytotoxicity. Hum Immunol. (2017) 78:103–12. 10.1016/j.humimm.2016.11.006 27884732

[34] Dons'koi BV Osypchuk DV Chernyshov PV. Enumeration of peripheral blood NKp46 positive NK lymphocytes reflects NK cytotoxic activity *in vitro*. J Immunol Methods. (2019) 474:112639. 10.1016/j.jim.2019.112639 31404551

[35] Hamzaoui K Berraies A Kaabachi W Ammar J Hamzaoui A. Pulmonary manifestations in Behcet disease: impaired natural killer cells activity. Multidiscip Respir Med. (2013) 8:29. 10.1186/2049-6958-8-29 23556512 PMC3637264

[36] Grom AA Villanueva J Lee S Goldmuntz EA Passo MH Filipovich A. Natural killer cell dysfunction in patients with systemic-onset juvenile rheumatoid arthritis and macrophage activation syndrome. J Pediatr. (2003) 142:292–6. 10.1067/mpd.2003.110 12640378

[37] Izumi Y Ida H Huang M Iwanaga N Tanaka F Aratake K. Characterization of peripheral natural killer cells in primary Sjogren's syndrome: impaired NK cell activity and low NK cell number. J Lab Clin Med. (2006) 147:242–9. 10.1016/j.lab.2006.01.001 16697772

[38] Hoechst B Voigtlaender T Ormandy L Gamrekelashvili J Zhao F Wedemeyer H. Myeloid derived suppressor cells inhibit natural killer cells in patients with hepatocellular carcinoma via the NKp30 receptor. Hepatology. (2009) 50:799–807. 10.1002/hep.23054 19551844 PMC6357774

[39] Cai L Zhang Z Zhou L Wang H Fu J Zhang S. Functional impairment in circulating and intrahepatic NK cells and relative mechanism in hepatocellular carcinoma patients. Clin Immunol. (2008) 129:428–37. 10.1016/j.clim.2008.08.012 18824414

[40] Jinushi M Takehara T Tatsumi T Hiramatsu N Sakamori R Yamaguchi S. Impairment of natural killer cell and dendritic cell functions by the soluble form of MHC class I-related chain A in advanced human hepatocellular carcinomas. J Hepatol. (2005) 43:1013–20. 10.1016/j.jhep.2005.05.026 16168521

