# Supplementary figures and images for: A Flow Cytometry-Based Whole Blood Natural Killer Cell Cytotoxicity Assay Using Overnight Cytokine Activation
Source: Front Immunol. 2020 Aug 14;11:1851. doi: 10.3389/fimmu.2020.01851 (PMC7457041; doi:10.3389/fimmu.2020.01851)

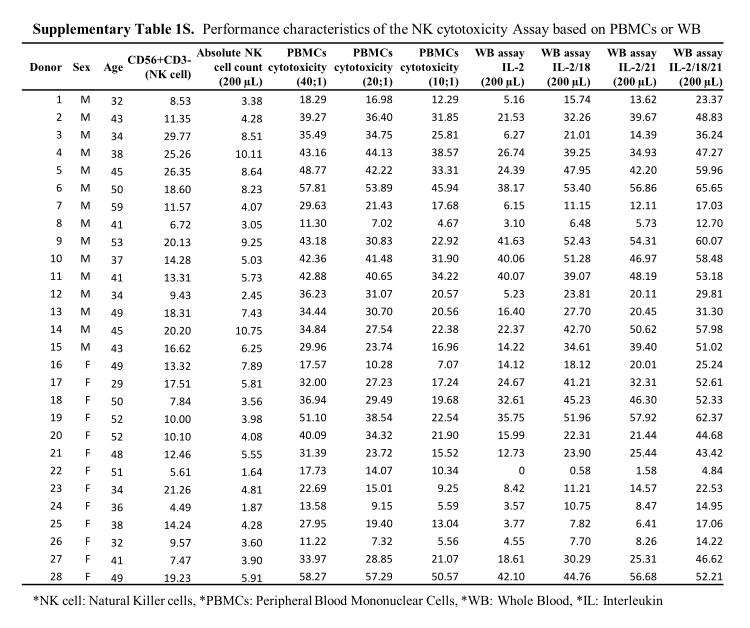

Supplement: Figure S1 — Comparison of natural killer (NK) cytotoxicity in hepatocellular carcinoma (n = 19, red) and liver cirrhosis (n = 7, blue) patients using whole blood (WB)-based NK cytotoxicity (200 μL). WB was activated overnight with IL-2, IL-2/IL-18, and IL-2/IL-18/IL-21. Data are represented as the mean ± SEM. [file Image_1.TIF]

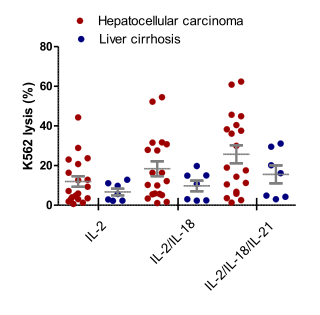

Supplement: Table S1 — Performance characteristics of the NK cytotoxicity assay based on PBMCs or WB. [file Image_2.TIF]
